# Deep learning to predict left ventricular hypertrophy from the electrocardiogram

**DOI:** 10.1093/europace/euag015

**Published:** 2026-01-23

**Authors:** Hafiz Naderi, Thomas Kaplan, Stefan van Duijvenboden, Esmeralda Ruiz Pujadas, Nay Aung, C Anwar A Chahal, Karim Lekadir, Bishwas Chamling, Marcus Dörr, Marcello R P Markus, Steffen E Petersen, Julia Ramírez, Patricia B Munroe

**Affiliations:** William Harvey Research Institute, Queen Mary University of London, Charterhouse Square, London, EC1M 6BQ, UK; Barts Heart Centre, St Bartholomew’s Hospital, Barts Health NHS Trust, West Smithfield, London, UK; William Harvey Research Institute, Queen Mary University of London, Charterhouse Square, London, EC1M 6BQ, UK; William Harvey Research Institute, Queen Mary University of London, Charterhouse Square, London, EC1M 6BQ, UK; Big Data Institute, La Ka Shing Centre for Health Information and Discovery, University of Oxford, Oxford, UK; Faculty of Mathematics and Computer Science, University of Barcelona, Barcelona, Spain; William Harvey Research Institute, Queen Mary University of London, Charterhouse Square, London, EC1M 6BQ, UK; Barts Heart Centre, St Bartholomew’s Hospital, Barts Health NHS Trust, West Smithfield, London, UK; William Harvey Research Institute, Queen Mary University of London, Charterhouse Square, London, EC1M 6BQ, UK; Barts Heart Centre, St Bartholomew’s Hospital, Barts Health NHS Trust, West Smithfield, London, UK; Centre for Inherited Cardiovascular Diseases, WellSpan Health, Lancaster, PA, USA; Department of Cardiovascular Diseases, Mayo Clinic, Rochester, MN, USA; Faculty of Mathematics and Computer Science, University of Barcelona, Barcelona, Spain; Institució Catalana de Recerca i Estudis Avançats (ICREA), Barcelona, Spain; Department of Internal Medicine B, University Medicine Greifswald, Greifswald, Germany; German Center for Cardiovascular Research (DZHK), Partner Site Greifswald, Greifswald, Germany; German Center for Diabetes Research (DZD), Partner Site Greifswald, Greifswald, Germany; German Center for Cardiovascular Research (DZHK), Partner Site Greifswald, Greifswald, Germany; Institute for Community Medicine, SHIP/KEF, University Medicine Greifswald, Greifswald, Germany; Department of Internal Medicine B, University Medicine Greifswald, Greifswald, Germany; German Center for Cardiovascular Research (DZHK), Partner Site Greifswald, Greifswald, Germany; German Center for Diabetes Research (DZD), Partner Site Greifswald, Greifswald, Germany; William Harvey Research Institute, Queen Mary University of London, Charterhouse Square, London, EC1M 6BQ, UK; Barts Heart Centre, St Bartholomew’s Hospital, Barts Health NHS Trust, West Smithfield, London, UK; William Harvey Research Institute, Queen Mary University of London, Charterhouse Square, London, EC1M 6BQ, UK; Aragón Institute of Engineering Research, University of Zaragoza, Zaragoza, Spain; Centro de Investigación Biomédica en Red—Biomateriales, Bioingeniería y Nanomedicina, Zaragoza, Spain; William Harvey Research Institute, Queen Mary University of London, Charterhouse Square, London, EC1M 6BQ, UK

**Keywords:** Left ventricular hypertrophy, Electrocardiogram, Deep learning, Machine learning

## Abstract

**Aims:**

Left ventricular hypertrophy (LVH) is a strong predictor of cardiovascular disease. We previously compared supervised machine learning techniques to classify cardiac magnetic resonance (CMR)-derived LVH using electrocardiogram (ECG) and clinical variables in 37 534 UK Biobank participants, obtaining an area under the receiving operating curve (AUROC) of 0.85, but with limited specificity and requiring external validation. In this study, we develop a deep learning (DL) model to improve classification with external evaluation in the Study of Health in Pomerania (SHIP).

**Methods and results:**

We analysed 12-lead ECGs of 48 835 participants from the UK Biobank imaging study. The dataset was split into a training set (70%), validation set (15%), and test set (15%) for performance evaluation. The model architecture was a fully convolutional network, for which the input was the participants’ median ECG and clinical variables and the predicted indexed left ventricular mass (iLVM) as the output. A subsequent logistic regression model was used to recalibrate iLVM predictions. In UK Biobank, 717 (1.5%) participants had CMR-derived LVH and the AUROC for the DL model was 0.97. The ECG components most predictive of LVH were the QRS complex and ventricular rate. The DL model outperformed our supervised algorithms, previous DL modelling efforts and clinical ECG benchmarks. There was modest generalizability of the DL model to 1423 participants in SHIP (AUROC 0.78), with differences in clinical profile, ECG acquisition, and CMR labelling as important factors.

**Conclusion:**

Our findings support the feasibility of scalable DL-based screening tools for the prediction of LVH from the ECG, whilst highlighting the need for model development using larger datasets with greater diversity to ensure generalizability.

## Graphical Abstract

**Graphical Abstract:**
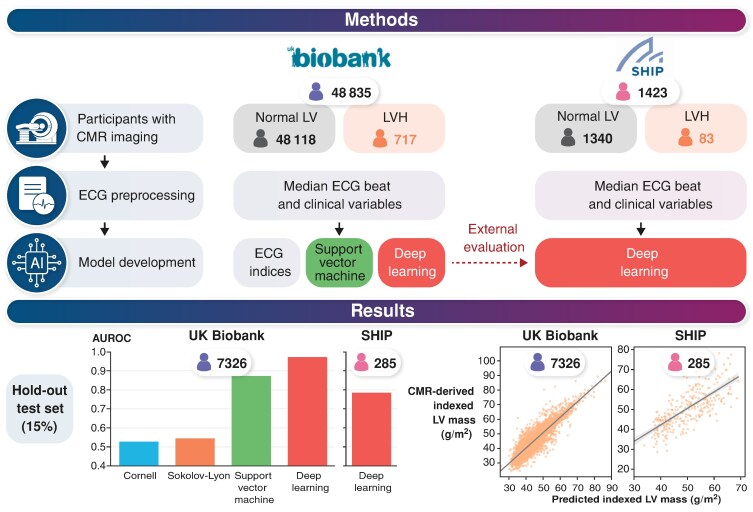

What’s new?

- In this study, we developed a fully convolutional deep learning (DL) model integrating 12-lead electrocardiogram (ECG) and clinical data to predict cardiac magnetic resonance-derived left ventricular hypertrophy (LVH) in UK Biobank (AUROC 0.97), outperforming our supervised models, previous DL-based efforts and conventional ECG criteria.
- Demonstrated modest but promising generalizability in external evaluation (AUROC 0.78), highlighting domain shift challenges.
- Our findings support the feasibility of developing scalable DL-based screening tools for the prediction of LVH from the ECG.

## Introduction

Left ventricular hypertrophy (LVH) is an established independent risk factor for adverse cardiovascular events.^1,2^ Early detection of LVH enables timely intervention and risk stratification, yet it remains underdiagnosed due to limitations in current diagnostic approaches.^3^ In clinical practice, the 12-lead electrocardiogram (ECG) is the most accessible and widely used diagnostic tool for detecting LVH. Despite its ubiquity, the ECG has limited sensitivity in identifying LVH when using conventional criteria such as the Sokolow–Lyon or Cornell voltage indices.^4,5^ Imaging modalities such as echocardiography and cardiac magnetic resonance (CMR) imaging offer more accurate structural characterization; however, they are resource-intensive and less feasible for large-scale screening.

In previous work, we compared supervised machine learning techniques to classify CMR-derived indexed left ventricular mass (iLVM).^6^ We showed that a set of 23 ECG biomarkers with physiological association with LVH, and clinical variables could classify LVH in 37 534 UK Biobank (UKB) participants with an area under the receiver operator curve (AUROC) of 0.85. These are promising results; however, there remains room to enhance diagnostic accuracy for detecting LVH. Recent advances in machine learning have shown promise in augmenting ECG interpretation by uncovering features beyond human perception. Deep learning (DL) models have successfully been applied to identify a range of cardiovascular conditions directly from raw ECG waveforms, including impaired ejection fraction,^7^ atrial fibrillation,^8^ and hypertrophic cardiomyopathy.^9^ These methods leverage the rich information embedded in the ECG signal to detect subtle physiological signatures associated with structural heart disease, with the added potential to reveal unidentified ECG features. Studies using DL applied to the ECG for LVH prediction in UKB have been reported with modest diagnostic performance in earlier releases of the UKB imaging cohort, achieving a c-statistic of 0.65^10^ (*N* = 32 239) and an AUROC of 0.72^11^ (*N* = 38 686).

In this study, we explore agnostic approaches to improve LVH classification and develop a DL model to predict CMR-derived LVH from the 12-lead ECG and clinical variables using the updated UKB cohort (*N* = 48 835). We assess the model’s diagnostic performance by comparing our approach to previous studies using DL, our supervised machine learning methods and conventional ECG clinical benchmarks. We also evaluate the model’s performance in an external population cohort with CMR-derived iLVM annotations.

## Methods

This study adheres to the European Heart Rhythm Association (EHRA) AI checklist, which is provided in Supplementary data.^12^

### Sample selection

The primary sample used for model development and evaluation consisted of 48 835 participants from the baseline UKB imaging study with paired CMR and ECG data. Left ventricular hypertrophy was characterized using CMR parameters derived using an existing analysis pipeline,^13,14^ whereby DL models were trained to automatically annotate the LV myocardium and hence derive CMR parameters. The UKB dataset was split into a training set (70%), validation set (15%), and hold-out test set (15%) for performance evaluation. The key CMR parameter of interest was LVM. Indexing with respect to body surface area was performed with the Mostellar formula.^15^ Left ventricular hypertrophy was defined as iLVM > 70 g/m^2^ (males) and > 55 g/m^2^ (females) with respect to normal ranges published for the UKB imaging study,^13^ corresponding to the thresholds in which sex-specific iLVM exceeds the 95% prediction interval for at least one of their reference age groups. To assist in interpretations of our results, we derived potential causes of LVH in these participants (*Table 1*): Hypertension, based on diagnoses, medication, and measurements (described in ‘Clinical variables’ section); hypertrophic cardiomyopathy, as the presence of rare coding variants (minor allele frequency < 0.00004) in eight implicated genes using whole exome sequence data and potential phenocopies (Fabry disease, amyloidosis, glycogen storage diseases, and RSAopathies).^16,17^

**Table 1:** Baseline characteristics of the UKB and SHIP participants

	UKB (*N* *=* 48 835)				SHIP (*N* *=* 1423)				*P*		
	All (*N* = 48 835)	LVH (*N* = 717)	Normal LV (*N* = 48 118)	*P*	All (*N* = 1423)	LVH (*N* = 83)	Normal LV (*N* = 1340)	*P*	All	LVH	Normal
Age (years)	65 (7.8)	64 (7.7)	65 (7.8)	**0**.**04**	52 (13.2)	52 (11.9)	52 (13.3)	0.8	**<0.001**	**<0.001**	**<0.001**
Sex, female (%)	25 315 (51.8)	353 (49.2)	24 962 (51.9)	**0**.**02**	653 (45.9)	32 (38.6)	621 (46.3)	0.2	**<0.001**	0.1	**<0.001**
BMI (kg/m^2^)	26.0 (4.3)	26.84 (4.9)	25.99 (4.3)	**<0.001**	26.94 (4.2)	27.75 (4.3)	26.89 (4.2)	0.2	**<0.001**	0.6	**<0.001**
Ethnicity, White European (%)	47 220 (96.7)	690 (96.2)	46 530 (94.4)	0.5	1 423 (100.0)	83 (100.0)	1 340 (100.0)		**<0.001**	0.1	**<0.001**
Systolic BP (mmHg)	142.5 (21.2)	159.0 (23.0)	142.0 (21.1)	**<0.001**	127.5 (17.6)	134.0 (18.7)	127.0 (17.3)	**<0.001**	**<0.001**	**<0.001**	**<0.001**
Diastolic BP (mmHg)	81.0 (11.4)	86.00 (13.1)	81.00 (11.3)	**<0.001**	78.0 (10.2)	82.0 (11.5)	77.8 (10.1)	**0**.**01**	**<0.001**	**0**.**001**	**<0.001**
Potential causes of LVH (%)											
*Hypertension*	35 903 (73.5)	620 (86.5)	35 283 (73.3)	**<0.001**	917 (64.4)	67 (80.7)	850 (63.4)	**0**.**001**	**<0.001**	0.1	**<0.001**
*HCM variant carrier*	5281 (10.8)	88 (12.3)	5193 (10.8)	**<0.001**							
*Phenocopies*	30 (0.1)	0 (0.0)	30 (0.1)								
High cholesterol (%)	31 388 (64.3)	456 (63.6)	30 923 (64.3)	0.7	1 039 (73.0)	60 (72.3)	979 (73.1)	0.9	**<0.001**	0.2	**<0.001**
*Total cholesterol* (mmol/L)	4.9 (1.2)	4.9 (1.1)	4.96 (1.2)	**0**.**02**	5.4 (1.1)	5.50 (1.2)	5.40 (1.1)	0.5	**<0.001**	**<0.001**	**<0.001**
*Non-HDL cholesterol* (mmol/L)	3.5 (1.2)	3.5 (1.1)	3.5 (1.2)	0.1	3.9 (1.1)	4.06 (1.2)	3.97 (1.1)	0.9	**<0.001**	**0**.**003**	**<0.001**
Diabetes (%)	2 738 (5.6)	57 (7.9)	2 681 (5.6)	**0**.**01**	104 (7.3)	9 (10.8)	95 (7.1)	0.1	**0.01**	0.4	**0.02**
Smoking status (%)											
*Never*	29 780 (61.0)	419 (58.4)	29 361 (61.0)	0.2	0 (0)	0 (0)	0 (0)		**<0.001**	**<0.001**	**<0.001**
*Previous*	16 360 (33.5)	238 (33.2)	16 122 (33.5)	0.9	1 118 (78.6)	60 (72.3)	1058 (78.9)	0.2	**<0.001**	**<0.001**	**<0.001**
*Current*	2 695 (5.5)	60 (8.4)	2 635 (5.5)	**0**.**002**	304 (21.4)	23 (27.7)	281 (20.9)	0.2	**<0.001**	**<0.001**	**<0.001**
Alcohol intake status (%)				0.9					0.6	0.8	0.54
*Never*	2 578 (5.3)	36 (5.0)	2 542 (5.3)		79 (5.5)	3 (3.6)	76 (5.7)	0.6			
*Current*	46 257 (94.7)	681 (94.9)	45 576 (94.7)		1 344 (94.5)	80 (96.4)	1 264 (94.3)	0.6			
Ventricular rate (beats/min)	61.7 (10.2)	59.3 (10.8)	61.7 (10.2)	**<0.001**	63.3 (10.4)	61.48 (9.9)	63.29 (10.5)	**0**.**03**	**<0.001**	0.1	**<0.001**
LVM (*g*)	82.5 (22.2)	131.4 (32.3)	82.0 (21.2)	**<0.001**	96.3 (26.2)	145.25 (28.6)	93.82 (24.1)	**<0.001**	**<0.001**	0.2	**<0.001**
Indexed LVM (g/m^2^)	44.0 (8.4)	70.4 (10.5)	43.8 (7.9)	**<0.001**	49.3 (9.9)	71.72 (9.2)	48.34 (8.8)	**<0.001**	**<0.001**	0.5	**<0.001**

BMI: body mass index, BP: blood pressure, HCM: hypertrophic cardiomyopathy, HDL: high-density lipoprotein, LVH: left ventricular hypertrophy, LVM: left ventricular mass, SHIP: Study of Health in Pomerania, UKB: UK Biobank.

### ECG processing

A 12-lead ECG was performed for participants of the UKB imaging study on the same day as the CMR imaging. We analysed the median heartbeat waveform across eight independent ECG leads (I, II, and V1–6), derived from the raw 15 s signals. The median beats were calculated by a classical method: initial bandpass Butterworth filtering between 1 and 45 Hz; peak-picking to identify R waves from the ECG principal components; beat alignment using time-lagged cross-correlation, with filtering of uncorrelated beats; and final averaging of retained beat waveforms. Given the use of a signal-averaged ECG waveform, the mean R-R interval (ventricular rate) was included as the only ECG biomarker in participant metadata (i.e. alongside the clinical variables defined in the following section).

### Clinical variables

Several clinical variables associated with LVH were included as metadata (*Table 1*). Clinical variables were derived using a combination of self-reported questionnaires performed at the imaging assessment visit, formal diagnoses and medications linked from primary care, physical measures, and biochemistry. Disease associations included hypertension, hypercholesterolaemia, and diabetes mellitus. Blood pressure (BP) measurements were averaged across readings taken at the imaging assessment centre visit. If participants were taking BP-lowering medication, their averaged (manual) BP readings were adjusted by adding either 15 mmHg to systolic BP or 10 mmHg to diastolic BP as per previous work.^18^ Hypertension was further defined based on formal diagnoses and the use of BP medication, or BP levels exceeding a 130/85 mmHg cut-off. Diabetes mellitus was determined by haemoglobin A1c (HbA1c) ≥ 48 mmol/mol. Hypercholesterolaemia was defined by serum total cholesterol of ≥ 5 mmol/L, having corrected for cholesterol-lowering medication by dividing total and non-HDL cholesterol by 0.73 and 0.66, respectively.^19^ As noted in the previous section, the mean R-R interval was included as a sole ECG biomarker alongside other clinical variables.

### Model architecture

We evaluated a performant network architecture for time series classification, a Fully Convolutional Network (FCN).^20^ The FCN serves as an effective baseline architecture that has been demonstrated to perform accurate classification across a range of multivariate time series datasets, even compared with state-of-the-art approaches.^21^ Our FCN consisted of three convolutional blocks, but convolutional parameters and operations included in each block were selected through hyperparameter optimization (described in ‘Training framework’ section). As part of this optimization, we evaluated the inclusion of max pooling layers to reduce overfitting, and batch normalization to speed up convergence whilst improving model generalization. A global average pooling (GAP) layer was used after the convolutions, drastically reducing the number of weights (parameters) used to represent the ECG features. The GAP output is concatenated with participant metadata before passing through two fully connected layers, which were also parameterized through optimization.

In addition to the FCN, we evaluated an open-source Residual Network (ResNet) as the most performant architecture of many for a related task—classifying hypertrophic cardiomyopathy for participants with hypertension.^22^ We evaluated their optimal configuration, a large 34-layer ResNet (ResNet34), to assess the potential performance implication of using a larger DL architecture (∼7.5 M parameters as opposed to ∼ 293k), including common optimizations for ECG modelling (residual connections).^23^ The ResNet34 was modified similarly to the FCN to include an output head for which the participant metadata was concatenated with ECG features output by the convolutional blocks. The model was implemented using Python v3.11.11 and PyTorch v2.6.0 (CPU-only). The versions of other Python dependencies are made available via the linked code repository.^24,25^

### Model configurations

We trained separate models to predict CMR-derived LVH (binary classification) and iLVM (g/m^2^); and henceforth, when referring to LVM, we will be referring to the indexed version of the measurement. Models were named based on their target variable, FCN_LVH_ and ResNet34_LVH_ for binary classification of CMR-derived LVH from the ECG, or FCN_LVM_ and ResNet34_LVM_ for regression over CMR-derived LVM from the ECG. Additionally, variants of each model were trained with and without inclusion of participant metadata (i.e. clinical variables), such that the output heads of each model consisted solely of fully connected layers with ECG features as input—it was previously found that clinical metadata did not improve DL discrimination of LVH within UKB.^10^

### Preprocessing

Median ECG waveforms were transformed to millivolts (mV) to shift the distribution closer to that of a unit interval. Continuous-valued features were scaled and translated into a unit interval relative to the training partition of the UKB cohort (min-max normalization). The smoking status variable was re-encoded in multiple columns for each of the possible status values (one-hot encoding), whereas other categorical variables were binary and unchanged.

### Training framework

The UKB cohort’s validation split was used to determine when the model performance had stopped improving, with an early stopping criterion of 20 epochs without improvement, and learning rate reduction by a factor of 0.1 when the model had not improved within 10 epochs. For all model configurations analysed, training terminated within 200 epochs. To reduce sensitivity to errors from outliers, we used the logarithm of hyperbolic cosine (log-cosh) as a loss function in predicting LVM, i.e. improving robustness of training given the long-tailed LVM distribution. Hyperparameter optimization was performed for the FCN architecture using a variant of the Hyperband algorithm (Tune).^26^ Optimizations for the learning process were the optimizer used (Adam or stochastic gradient descent), learning rate {1e-4, 5e-4, 1e-3, 1e-2} and batch size {16, 32, 64, 128}. The selected training parameters resulting in optimal validation losses were the Adam optimizer with a learning rate of 5e-4 and batch size of 64.

**Figure 1:**
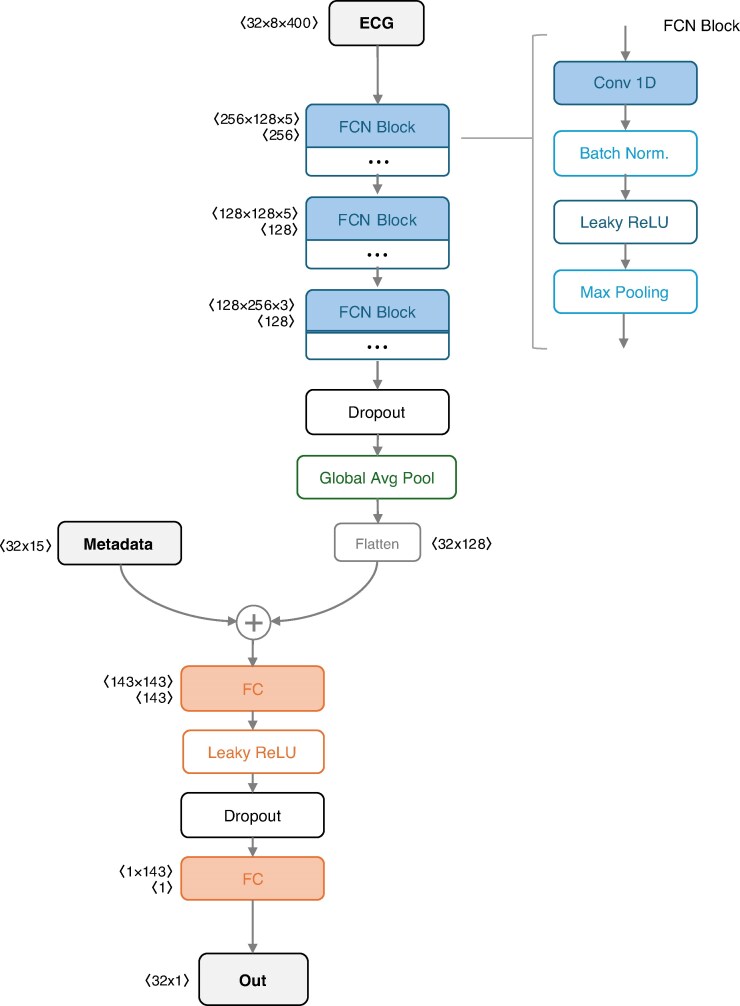
Fully convolutional network (Wang et al., 2016) architecture used in the present work, consisting of three convolutional blocks, the output of which is pooled and concatenated with clinical metadata and output through two fully connected layers for final predictions.

Optimizations for the FCN included the general convolution configuration (filter count and length), which either followed the configuration proposed by Wang et al. (2016) or a smaller but compatible configuration used by Zhou et al. (2024) for encoding similar median ECG waveforms from UKB.^20,27^ We additionally optimized for: use of batch-normalization within convolutional blocks; use of max pooling within convolutional blocks; dropout after the convolutional layer (probability *P* = 0, 0.1, …, 0.8); dropout after the first fully connected layer (probability *P* = 0, 0.1, …, 0.8); and the inclusion of metadata. The selected configuration resulting in optimal validation losses was that of Wang et al. (2016), with the addition of batch normalization and max-pooling layers within each convolutional block; a dropout of 0.4 after the convolutional layers; a dropout of 0.6 between the fully connected layers; and the inclusion of metadata.^20^ The FCN network architecture is illustrated in *Figure 1*.

### Left ventricular hypertrophy classification

Further to the FCN_LVH_, which directly classified participants with LVH or normal LV mass, iLVM predictions output by the FCN_LVM_ were used to derive LVH cases in two ways. First, using the iLVM cut-offs previously specified. Secondly, iLVM predictions were inputted into a separate logistic regression (LR) model, which learned to classify instances of LVH, allowing for custom decision thresholds (in terms of iLVM) that diverge from published ranges, i.e. a linear recalibration that compensates for systematically skewed predictions reflecting the imbalanced ground-truth iLVM distribution. Sex was also input to the LR as a single covariate, given the significant iLVM difference between sexes according to reference ranges derived from CMR in UKB.^13^ Together, the FCN and LR (FCN_LVM_ + LR) pipeline provides an opaque LVH classification that can be compared with the other two methods. Sample weights for the LR model were balanced according to class frequencies to account for the class imbalance between LVH and normal LV cases.

### Performance benchmarks

As baseline performance benchmarks, we used our previous supervised methods from the existing literature for the classification of LVH, which outperformed the only existing DL approach for LVH classification within the UKB cohort.^6,10^ In addition to the aforementioned clinical variables, these supervised methods were trained using an extensive set of ECG biomarkers (e.g. QRS duration, QRS wave amplitude, and pathological Q waves) extracted automatically using signal processing. The most performant model was an optimized support vector machine (SVM), using a radial basis function kernel with a regularization constant *C* = 1. This was re-implemented and trained on identical splits of our present dataset, using random under-sampling consistently to balance against the minority class (LVH). The original ECG feature extraction pipeline used was run on the ECGs that were not part of their original cohort. The same cohort splits were used to train the SVM. Notably, the validation set was used to perform a five-fold cross-validation grid search over hyperparameters, to ensure it was not possible to identify an updated parameter set outperforming that selected in the original work. We also evaluated two clinically used ECG criteria for LVH, calculated with the ECG biomarkers extracted using the pipeline noted above: Sokolow–Lyon and Cornell voltage.^28,29^

### External evaluation

The Study of Health in Pomerania (SHIP) was used for the external evaluation of the models trained in UKB. Study of Health in Pomerania is a study investigating common risk factors in a random sample of the population from West Pomerania, Northeastern Germany.^30^ A total of 1474 participants drawn from the baseline SHIP-TREND-0 cohort and second follow-up SHIP-START-2 cohorts were studied in the present work, given the availability of paired CMR and 12-lead ECG data. Electrocardiograms were processed from EDF files, using PyEDFlib, before processing consistent with that of the UKB ECGs.^31^ There were only six missing fields across a total of four participants in SHIP, which were imputed using either mode or median imputation for binary or continuous columns, respectively: two systolic BP, two diastolic BP, one total cholesterol level, and one smoking status.

Given the limited UKB sample available for model training and the differing cohort characteristics compared with SHIP (*Table 1*), the DL models pre-trained on UKB data were fine-tuned in SHIP to mitigate domain shift effects and improve generalizability.^32^ The SHIP dataset was split into a training set (60%), validation set (20%), and hold-out test set (20%) for performance evaluation. Given the small size of the cohort, we performed random data augmentation for the training set: random crops at the start and end of all leads (uniform sampling of up to 25 samples for each end), followed by linear interpolation; random Gaussian noise per lead (*M* = 0, SD = 0.005); and minor amplitude scaling across leads (uniform sampling between [0.9, 1.1]). The augmentations used were selected to preserve ECG morphology. The same optimizer (Adam) was used for fine-tuning, with additional parameter weight-decay (regularization) to reduce overfitting. Layers were incrementally unfrozen for fine-tuning, starting with the output heads (learning rate 5e-5, weight-decay 1e-4), and then each convolutional block (learning rate 1e-4, weight-decay 1e-5).

### Statistical analyses

For classification performance analyses, confidence intervals for AUROC were calculated analytically.^33,34^ Significant differences in AUROC were calculated using a fast implementation of DeLong’s algorithm.^35^ Sensitivity and specificity were reported at operating points where the difference between TPR and FPR (Youden’s J statistic) is maximal, i.e. an optimized decision threshold. Regression performance was assessed using the Pearson correlation coefficient, linear regression using the ordinary least squares (OLS) method and Bland–Altman agreement. Accuracy was reported in terms of mean absolute error (MAE) and mean error (ME) with 95% confidence intervals computed via bootstrapping (*N* = 5000).

### Model explainability

The importance of ECG regions and clinical variables to model predictions was assessed using the Integrated Gradients feature attribution method, as implemented in the SHapley Additive exPlanations package (SHAP, v0.47.1).^36,37^ Integrated Gradients resemble SHAP values but use the gradients operator of a deep neural network to identify salient input features with respect to some background (baseline) sample—for which we use a large random sampling of *N* = 1000 participants’ ECGs and clinical metadata to avoid possible instability of the analyses.^38^ Put simply, integrated gradients SHAP evaluates how sensitive the DL model predictions are to changes in the input features. We report feature importances at a local level, for participants with predicted iLVM below or above the 5th and 95th percentiles, respectively, i.e. extremes of both measures, to illustrate the most informative clinical variables and ECG features. Whilst the method was applied at a local level, it also offers individual-level explainability (e.g. saliency of an individual ECG waveform).

## Results

### Study populations

The characteristics of the UKB participants (*N* = 48 835) and the external evaluation cohort (*N* = 1423), the Study of Health in Pomerania (SHIP), are shown in *Table 1*. Compared to UKB, the SHIP cohort was younger (mean age 65 vs. 52 years, *P* < 0.001), had a slightly lower proportion of females (52% vs. 46%, *P* < 0.001) and a higher prevalence of LVH (1.5% vs. 5.8%, *P* < 0.001) and higher overall iLVM (44 g/m^2^ vs. 49 g/m^2^, *P* < 0.001). The most common potential cause of LVH in participants was hypertension (73.5% in UKB and 64.4% in SHIP). A relatively small portion of UKB participants carried rare coding variants for genes implicated in hypertrophic cardiomyopathy (10.8%), with a marginal but significantly higher prevalence in individuals with LVH (12.3% vs. 10.8%, *P* < 0.001). Very few participants were identified with potential phenocopies (*N* = 30, 0.1%).

### Left ventricular mass prediction

**Figure 2:**
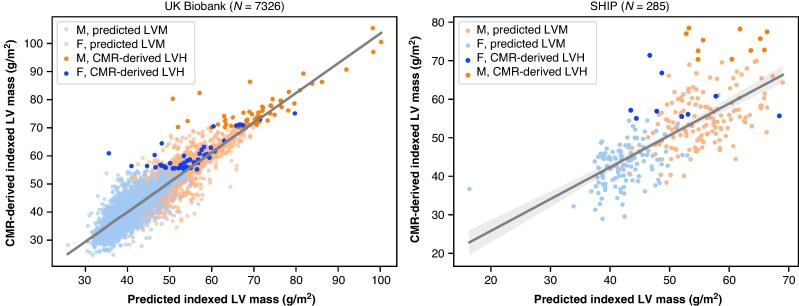
Correlation between predicted indexed LVM and CMR-derived indexed LVM in UKB and SHIP test sets. Diagonal lines are linear OLS fits with shaded confidence intervals (95%). CMR, cardiac magnetic resonance; LVM, left ventricular mass; OLS, ordinary least squares; SHIP, Study of Health in Pomerania; UKB, UK Biobank.

**Figure 3:**
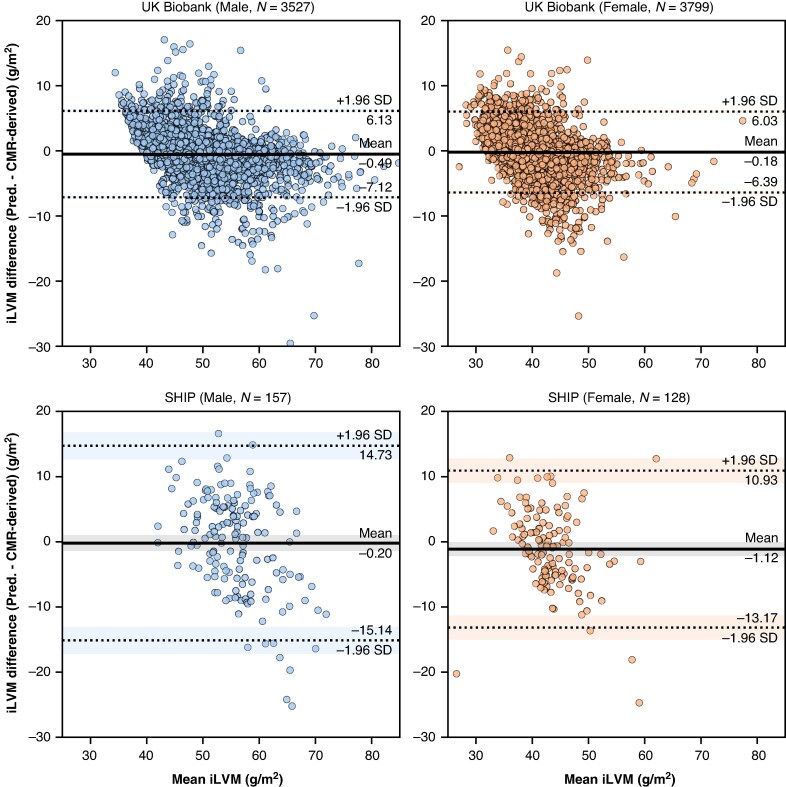
Bland–Altman plots demonstrating pairwise agreement of predicted indexed LVM and CMR-derived LVM in UKB and SHIP test sets, by sex. Horizontal dashed lines indicate upper and low limits of agreement (95%) and the respective shaded confidence intervals (95%). CMR, cardiac magnetic resonance; LVM, left ventricular mass; SHIP, Study of Health in Pomerania; UKB, UK Biobank.

Indexed left ventricular mass predictions were accurate (MAE 2.21 g/m^2^ [2.16, 2.27]) and had a strong correlation with CMR-derived LVM (adj. *R*^2^ = 0.85, *P* < 0.001; *r* = 0.92, *P* < 0.001). *Figure 2* reveals that differences in CMR-derived and predicted iLVM were more pronounced in several of the participants with LVH. Generally, iLVM was systematically underestimated (ME −0.33 g/m^2^ [−0.26, −0.40], skew = −0.41), reflecting the imbalanced CMR-derived iLVM distribution whereby a relatively small sub-population has LVH. Bland–Altman agreement in *Figure 3* better illustrates the heteroscedasticity of iLVM predictions, with a fan-like pattern indicating increasing prediction error at the upper extremes of iLVM (i.e. LVH).

### Left ventricular hypertrophy classification

LVH classification performance is reported in *Table 2*. In terms of AUROC, classification performance was greatest in the FCN_LVM_ + LR (AUROC = 0.97; 95% confidence interval 0.95–0.99), significantly outperforming the SVM model (AUROC = 0.87 [0.84–0.90]). This largely stemmed from improved specificity in the FCN_LVM_ + LR (specificity = 0.95 [0.95–0.96]) compared to the SVM (specificity = 0.75 [0.74–0.76]), despite more comparable sensitivity between the FCN_LVM_ + LR (sensitivity = 0.92 [0.85–0.95]) and SVM (sensitivity = 0.87 [0.79–0.92]). The FCN_LVM_ recorded the highest specificity (specificity = 0.99 [0.99–1.00]), but it did not achieve an AUROC improvement over the SVM, as the LVH diagnoses derived from the pre-determined threshold on predicted iLVM lacked sensitivity due to systematic underestimation. Accordingly, the LR-optimized decision thresholds are shown in Supplementary material online, *Figure S1* (>58.9 g/m^2^ for males and > 46.9 g/m^2^ for females), indicating a slight revision of LVH cut-offs compared to our previous publication.^13^ The FCN variant directly predicting LVH, FCN_LVH_, achieved a modest diagnostic improvement compared to the SVM. The AUROC curves and optimized operating points are shown in Supplementary material online, *Figure S2*. The AUROC differences between the SVM and FCN variants were statistically significant, DeLong’s test *P* < 0.001. Both the SVM and FCNs saw dramatic sensitivity improvements compared to the classical ECG criteria, which were limited for both Sokolow–Lyon (sensitivity = 0.10 [0.06–0.18]) and Cornell voltage (sensitivity = 0.12 [0.07–0.20]) criteria. Support vector machine and FCN variants depend entirely on the ECG (i.e. excluding clinical features) performed comparably to those using both ECG and clinical features (see Supplementary material online, *Table S1*).

**Table 2:** LVH classification performance in UKB the FCN variants (present work), SVM (replication of Naderi et al., 2023), criteria for Sokolow–Lyon and Cornell voltage; with 95% confidence intervals in brackets

Model	AUROC	Sensitivity	Specificity	F1
FCN_LVH_	0.88 (0.85, 0.92)	0.81 (0.73, 0.88)	0.84 (0.83, 0.84)	0.52 (0.50, 0.54)
FCN_LVM_	0.73 (0.68, 0.78)	0.46 (0.37, 0.56)	0.99 (0.99, 1.0)	0.81 (0.79, 0.83)
FCN_LVM_ + LR	0.97 (0.95, 0.99)	0.92 (0.85, 0.95)	0.95 (0.95, 0.96)	0.66 (0.64, 0.68)
SVM	0.87 (0.84, 0.90)	0.87 (0.79, 0.92)	0.75 (0.74, 0.76)	0.47 (0.45, 0.49)
Sokolow–Lyon	0.54 (0.52, 0.57)	0.10 (0.06, 0.18)	0.98 (0.98, 0.99)	0.54 (0.52, 0.56)
Cornell voltage	0.52 (0.49, 0.56)	0.12 (0.07, 0.20)	0.92 (0.92, 0.93)	0.50 (0.48, 0.52)

AUROC: area under the receiver operator curve, FCN: Fully Convolutional Network, LR: logistic regression, LVH: left ventricular hypertrophy, LVM: left ventricular mass, SVM: support vector machine, UKB: UK Biobank.

### ECG saliency and feature importance

**Figure 4:**
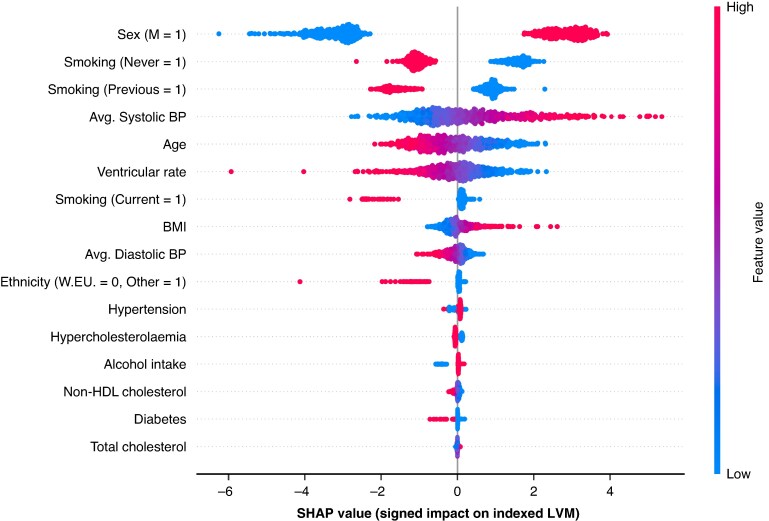
SHAP feature importance for participants’ clinical variables in the UKB test set. Magnitude of SHAP values (x-axis) corresponds to the prediction effect sizes, and direction to increased or decreased prediction values. Colors indicate the variable correlation, higher variable values in red and lower in blue. Binary value encodings are indicated on the label (y-axis), e.g. for sex, male (M) = 1, so shown in red—note the positive SHAP values, larger LVM predictions. LVM, left ventricular mass; SHAP, SHapley Additive exPlanations.

**Figure 5:**
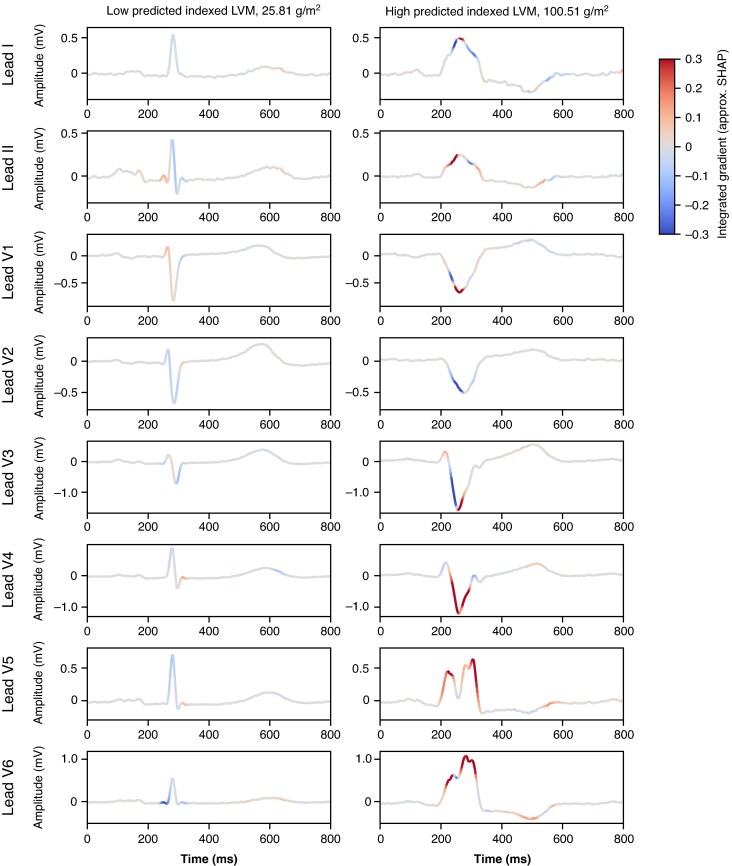
Approximated SHAP values (integrated gradients) for two participants’ median ECGs from the UKB test set, with low (left) and high (right) predicted indexed LVM respectively. Colours correspond to morphology effect, red increasing predictions and blue decreasing predictions, and grey having little relative effect. ECG, electrocardiogram; LVM, left ventricular mass; SHAP, SHapley Additive exPlanations; UKB, UK Biobank.

**Figure 6:**
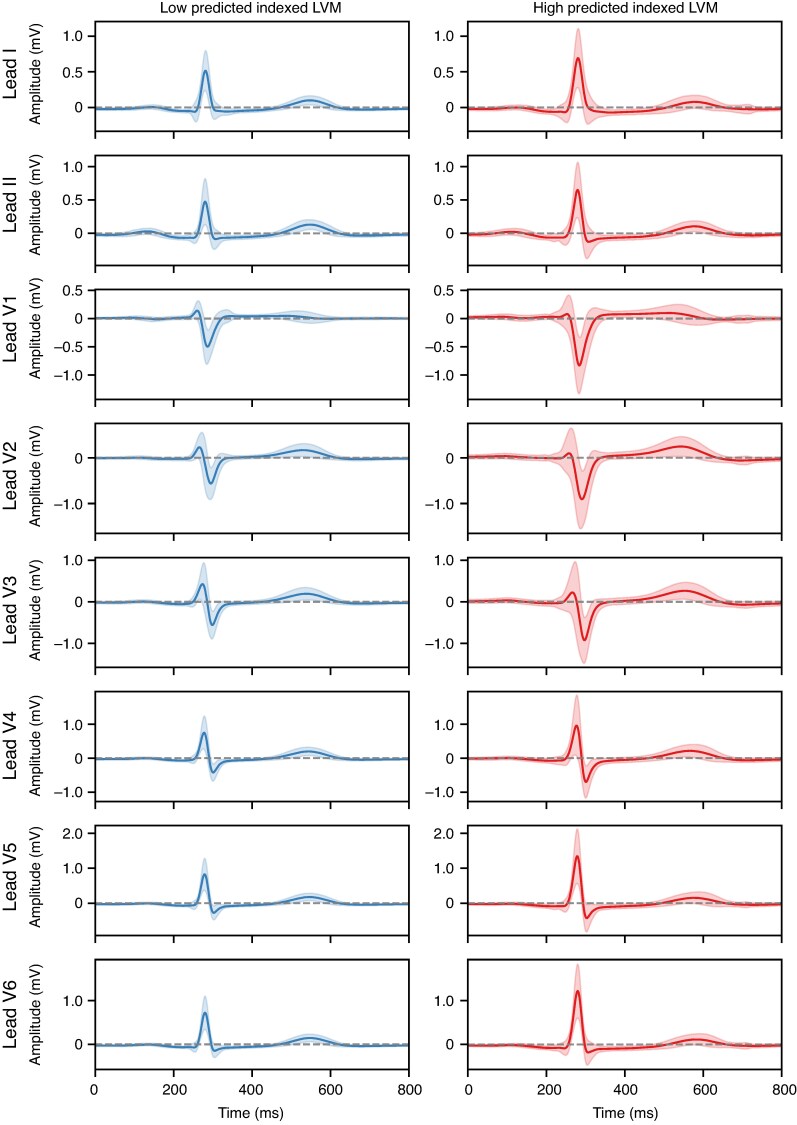
Mean ECG waveforms across participants from the UKB test set with low (below 5th percentile) or high (above 95th) percentile predicted indexed LVM. The shaded region corresponds to lower and upper SD bounds. ECG, electrocardiogram; LVM, left ventricular mass; SD, standard deviation; UKB, UK Biobank.

Clinical feature importance in terms of SHAP values is shown in *Figure 4* for participants from the UKB hold-out test partition. Sex, smoking status, and age were amongst the most predictive features for iLVM, in addition to ventricular rate (our only derived ECG feature), and systolic BP.^10,11^ Saliency maps for ECG waveforms (using approximated SHAP values) are shown in *Figure 5* for iLVM, for two examples of participants with low and high predicted iLVM by the FCN_LVM_. Similarly, mean ECGs for individuals with low and high predicted iLVM are shown in *Figure 6*. Generally, the components seemingly most relevant for LVM estimation are the QRS complex and *P*-wave.

### External evaluation in SHIP

**Figure 7:**
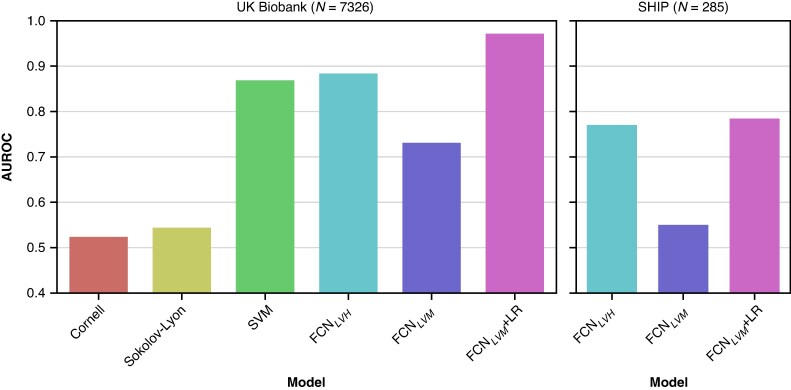
AUROC comparison in UKB and SHIP test sets. AUROC, area under the receiver operator curve, SHIP, Study of Health in Pomerania; UKB, UK Biobank.

The average iLVM prediction error increased (MAE 5.51 g/m^2^ [5.02, 6.03]), but remained relatively low in absolute terms, with a moderate correlation to CMR-derived iLVM (adj. *R*^2^ = 0.50, *P* < 0.001, *r* = 0.71, *P* < 0.001). Similarly to the UKB test set, iLVM was underestimated to a greater extent (ME 0.62 g/m^2^ [−0.20, 1.43], skew = −0.56). The corresponding correlations are shown in *Figure 2*. Left ventricular hypertrophy detection was moderate (*Table 3*), with a large decrease in AUROC for all FCN configurations, most notably the FCN_LVM_ + LR (AUROC = 0.78 [0.69, 0.88]), which performed more comparably to the FCN_LVH_ (AUROC = 0.78 [0.63, 0.93]), but the relative AUROC distribution for FCN variants in SHIP was broadly similar to that of the UKB test set (*Figure 7*).

**Table 3:** LVH classification performance in SHIP for FCN variants; with confidence intervals (95%) in brackets

Model	AUROC	Sensitivity	Specificity	F1
FCN_LVH_	0.78 (0.63, 0.93)	0.76 (0.53, 0.90)	0.85 (0.80, 0.89)	0.64 (0.54, 0.73)
FCN_LVM_	0.55 (0.48, 0.62)	0.10 (0.03, 0.30)	1.0 (0.99, 1.00)	0.57 (0.48, 0.62)
FCN_LVM_ + LR	0.78 (0.69, 0.88)	0.65 (0.43, 0.82)	0.80 (0.74, 0.84)	0.59 (0.49, 0.68)

AUROC: area under the receiver operator curve, FCN: Fully Convolutional Network, LR: logistic regression, LVH: left ventricular hypertrophy, LVM: left ventricular mass, SHIP: Study of Health in Pomerania.

## Discussion

In this study, we developed and evaluated a DL model to predict CMR-derived LVH using 12-lead ECG and clinical variables and demonstrated that the DL model outperformed prior machine learning methods and conventional ECG criteria in detecting LVH. External evaluation of the DL model in the SHIP cohort yielded modest generalizability, aligning with findings in other comparable studies, whereby external out-of-sample performance is typically limited.^10,11^

The DL model retained biological plausibility with SHAP analyses showing sex, age, and systolic BP being among the most influential clinical predictors, consistent with known risk factors for LVH. Importantly, the QRS complex and ventricular rate emerged as salient ECG components, supporting the pathophysiological underpinnings of LVH, which affects ventricular depolarization and conduction times.^5,39,40^ Clinically, resting heart rate can reflect cardiorespiratory fitness, autonomic tone, and haemodynamic compensation in the context of reduced diastolic filling time or reduced stroke volume with compensatory tachycardia.^41,42^ In addition, higher ventricular rates may act as a proxy for comorbidity burden that contributes to LV remodelling, including hypertension, obesity, and subclinical heart failure. Therefore, ventricular rate may represent a surrogate marker capturing correlated physiological stressors and disease phenotypes associated with LVH, rather than a direct mechanistic driver. Furthermore, the ECG-only configuration of DL models demonstrated robust performance, reinforcing the value of the raw ECG waveform for LVH prediction.

### Comparison to contemporary studies

There have been other contemporary studies that have also applied DL to ECG data for LVH prediction,^43,44^ and two using UKB.^10,11^ Khurshid et al. (2021) developed a 10-layer convolutional neural network (CNN) with residual connections to predict CMR-derived LVM from the ECG (entire 10 s) in 37 142 UKB participants with a c-statistic of 0.65 [0.61–0.70] (sensitivity = 0.34 [0.25, 0.44], specificity = 0.96 [0.96–0.97]), a similar predictive profile to that of the FCN_LVM_ in our present work.^10^ External validation was sought in 1371 patients from Mass General Brigham with a c-statistic of 0.62 [0.59–0.65] (sensitivity = 0.41 [0.36–0.46], specificity = 0.83 [0.80–0.86]), following a linear recalibration of LVM predictions with sex as a covariate. Radhakrishnan et al. (2023) developed a multi-modal model with separate CNNs for ECGs (1.2 s median beat) and MRIs, but a unified (cross-modal) latent space, allowing unimodal inference of several clinical phenotypes from the ECG alone in 38 686 UKB participants. In the case of LVH, they achieved an AUROC of 0.72 [0.70–0.73]. Our study outperformed these previous efforts whilst maintaining high sensitivity and specificity. Incorporating an LR step that recalibrated predictions using sex as a covariate significantly improved classification performance by adjusting decision thresholds, resembling the linear LVM recalibration step used by Khurshid et al. in external validation. This hybrid approach, which blends regression and classification methods, offered a flexible and interpretable mechanism for refining predictions in skewed populations. Notably, the use of an LR model, as opposed to a linear recalibration of iLVM predictions, offers the flexibility of an adaptive iLVM cut-off for LVH classification, accounting for distributional differences in iLVM predictions vs. CMR-derived iLVM. It is possible that reducing the dimensionality of the ECG waveform into a median beat, as opposed to analysing the entire 10-s waveform, simplified LVM prediction and enabled our use of a relatively small CNN (in terms of convolutional blocks) compared with previous studies. Analysing just a median beat might have also simplified training using the UKB sample, given the sample size is limited despite having increased in size since the previous studies, i.e. using the entire ECG might have necessitated a larger sample or pre-training. The modest generalizability of our model likely reflects domain shifts between cohorts, including demographic differences, ECG acquisition protocols, and downstream imaging analysis. Notably, the papillary muscles were excluded in the UKB CMR image analysis, in contrast to SHIP.^13,45^ These differing image analysis protocols may in part explain the prevalence of LVH in the cohorts and the DL model generalizability. Despite methodological considerations to address overfitting, it is possible that a degree of overfitting to the UKB sample (as the sole training dataset) also hindered generalization in SHIP. One promising approach to improve generalizability in future work will be to leverage foundation models with large-scale pre-training across millions of ECGs (not necessarily with paired CMR), which should rapidly adapt to specific tasks such as LVH detection in new datasets.^46–48^

Outside of the UKB, similar performance for LVH detection from ECGs has been reported in a few studies, but all in clinical cohorts and without external validation, making it challenging to directly compare approaches. Liu et al. (2023) studied ECGs from the Tri-service General Hospital Songshan Branch (Taipei, Taiwan), but their approach differed by using 24 derived ECG features as input to a small fully connected neural network (sensitivity = 0.97, specificity = 0.96).^49^ Kashou et al. (2020) studied a vast cohort from the Mayo Clinic ECG laboratory (*N* = 720 978), using a residual network with 33 convolutional layers (AUROC = 0.99, sensitivity = 0.96, and specificity = 0.94)^50^. Hughes et al. (2021) studied ECGs from the University of California, San Francisco, again using a large residual network (AUROC = 0.98, sensitivity = 0.97, and specificity = 0.85)^51^. These studies all report strong diagnostic performance comparable to that of our present study, but we have demonstrated that this does not guarantee generalization in an external cohort. Differences in training cohorts between our study and others might also lead to differing performance profiles dependent on external cohort characteristics, warranting validation in several cohorts. For example, training on a relatively healthy population cohort (UKB) might improve negative predictive value and diagnostic performance for borderline cases, whereas studies trained on higher-risk clinical cohorts might achieve greater sensitivity and accurately predict iLVM in ranges associated with LVH. This suggests that clinical translation of DL models for LVH screening requires training and calibration across both population and higher-risk clinical cohorts.

### Clinical utility

From a clinical perspective, the ECG is a widely accessible, low-cost diagnostic tool used routinely in practice. However, traditional ECG criteria for detecting LVH have limited sensitivity, making them sub-optimal for population-level screening. An artificial intelligence (AI)-enabled ECG model could support LVH detection as a scalable ‘front-line’ triage tool in settings where cardiac imaging capacity is constrained. For example, in primary care or hypertension clinics, a high-risk AI-ECG LVH score could prompt targeted confirmatory imaging (echocardiography or CMR), review of BP control and secondary causes, and closer follow-up. Conversely, a low-risk score could help deprioritize imaging in low-pre-test probability cases, reducing unnecessary investigations. In cardiology services, the model could be applied opportunistically to existing digital ECG archives to identify previously unrecognized patients who may benefit from risk factor optimization or evaluation for cardiomyopathy phenotypes. These potential workflows align with broader trends in digital cardiology where interoperable infrastructures, remote care pathways and scalable analytics are increasingly leveraged to triage large volume of longitudinal data and support more efficient, earlier risk identification, as highlighted in the recent EHRA summit.^52^ Future work should focus on prospective validation in diverse clinical settings, integration into electronic health records systems to enable automated flagging of high-risk individuals in routine care. It should also explore dynamic, risk-based thresholds that incorporate serial CGS and evolving clinical parameters. There is scope to integrate other features such as proteomics, metabolomics, biochemistry, and genetic risk scores to further personalize the model.

### Limitations

Our study has limitations that warrant discussion. Firstly, participants in UKB and SHIP are predominantly of white European ancestry and have limited data (especially SHIP); therefore, further training and validation across large and diverse ethnic populations with differing risk profiles would improve the fairness (inclusion) of future studies and might improve generalization and optimize performance across populations. This issue of participant homogeneity is evidenced by our fine-tuning of models trained in UKB as part of external evaluation in SHIP. Second, although we used a common method to adjust BP measurements for treatment effects from antihypertensive therapy,^18^ we acknowledge the limitation of uniform correction factors and the potential variability due to individual responses.

## Conclusions

The DL model integrating ECG and clinical variables effectively classified CMR-derived LVH, outperforming both previously developed supervised algorithms and current clinical ECG benchmarks. The model demonstrated moderate generalizability to an external community-based population, although differences in clinical characteristics and ECG acquisition methods impaired performance, emphasizing the need for model training across larger datasets with greater diversity ahead of further validation and broader deployment. Our findings support the feasibility of developing scalable, DL-based ECG screening tools for the prediction of LVH, whilst highlighting key considerations ahead of translation into a clinical setting.

### Ethics statement

This study complies with the Declaration of Helsinki; the work was covered by the ethical approval for UK Biobank studies from the NHS National Research Ethics Service on 17th June 2011 (Approval number 11/NW/0382) and extended on 18 June 2021 (Approval number 21/NW/0157) with written informed consent obtained from all participants. The work related to the Study of Health in Pomerania is via application reference number SHIP/2023/31/D. The study is covered by the overall ethical approval for SHIP studies approved by the Ethics Committee at the University Medicine Greifswald, Germany.

## Supplementary Material

euag015_Supplementary_Data

## Data Availability

The data underlying this article were provided by the UK Biobank under access application 2964. UK Biobank will make the data available to bona fide researchers for all types of health-related research that is in the public interest, without preferential or exclusive access for any persons. All researchers will be subject to the same application process and approval criteria as specified by UK Biobank. For more details on the access procedure, see the UK Biobank website: http://www.ukbiobank.ac.uk/register-apply/. Code for running the experiments, analysis and plotting is available on a Zenodo repository: https://github.com/Electrogenomics-Group/ai-ecg-lvh. This open-source resource is intended solely for research purposes and has not been approved for use by any legal authority.
