# Supplementary material for: Deep learning to predict left ventricular hypertrophy from the electrocardiogram
Source: Europace. 2026 Jan 23;28(2):euag015. doi: 10.1093/europace/euag015 (PMC12933001; doi:10.1093/europace/euag015)
Supplement: euag015_Supplementary_Data [file euag015_supplementary_data.pdf]

Supplementary Material

Supplementary Table 1. LVH classification in UKB for all model variants, including configurations only using the ECG (signal for FCN and ResNet, or features for SVM and ECG indices); with confidence intervals (95%) in brackets.

Model	Config	AUROC	Sensitivity	Specificity	F1
FCN_{LVH}	ECG + Clin	0.88 (0.85, 0.92)	0.81 (0.73, 0.88)	0.84 (0.83, 0.84)	0.52 (0.50, 0.54)
	ECG	0.86 (0.82, 0.90)	0.81 (0.72, 0.87)	0.79 (0.78, 0.80)	0.49 (0.47, 0.51)
FCN_{LVM}	ECG + Clin	0.73 (0.68, 0.78)	0.46 (0.37, 0.56)	0.99 (0.99, 1.00)	0.81 (0.79, 0.83)
	ECG	0.77 (0.72, 0.82)	0.54 (0.44, 0.63)	1.00 (1.00, 1.00)	0.83 (0.81, 0.85)
FCN_{LVM} + LR	ECG + Clin	0.97 (0.95, 0.99)	0.92 (0.85, 0.95)	0.95 (0.95, 0.96)	0.66 (0.64, 0.68)
	ECG†	0.97 (0.95, 0.99)	0.91 (0.84, 0.95)	0.94 (0.93, 0.94)	0.63 (0.61, 0.65)
$ResNet34_{LVH}$	ECG + Clin	0.83 (0.78, 0.87)	0.76 (0.67, 0.83)	0.76 (0.75, 0.77)	0.48 (0.46, 0.50)
	ECG	0.81 (0.77, 0.86)	0.67 (0.59, 0.77)	0.80 (0.79, 0.81)	0.49 (0.47, 0.51)
$ResNet34_{LVM}$	ECG + Clin	0.91 (0.87, 0.94)	0.80 (0.82, 0.87)	1.00 (1.00, 1.00)	0.94 (0.92, 0.95)
	ECG	0.91 (0.88, 0.95)	0.83 (0.75, 0.89)	1.00 (1.00, 1.00)	0.94 (0.93, 0.96)
$ResNet34_{LVM}$ + LR	ECG + Clin	0.97 (0.95, 0.99)	0.87 (0.81, 0.93)	0.97 (0.96, 0.97)	0.70 (0.68, 0.72)
	ECG†	0.97 (0.95, 0.99)	0.87 (0.79, 0.92)	0.99 (0.99, 0.99)	0.86 (0.84, 0.88)
SVM	ECG + Clin	0.87 (0.84, 0.90)	0.87 (0.79, 0.92)	0.75 (0.74, 0.76)	0.47 (0.45, 0.49)
	ECG	0.86 (0.82, 0.89)	0.81 (0.73, 0.88)	0.77 (0.76, 0.78)	0.48 (0.46, 0.50)
Sokolow-Lyon	-	0.54 (0.52, 0.57)	0.10 (0.06, 0.18)	0.98 (0.98, 0.99)	0.54 (0.52, 0.56)
Cornell voltage	-	0.52 (0.49, 0.56)	0.12 (0.07, 0.20)	0.92 (0.92, 0.93)	0.50 (0.48, 0.52)

† The LR was configured to use sex as a covariate in both configurations, i.e. include when the DL model used the ECG alone (and no clinical variables).

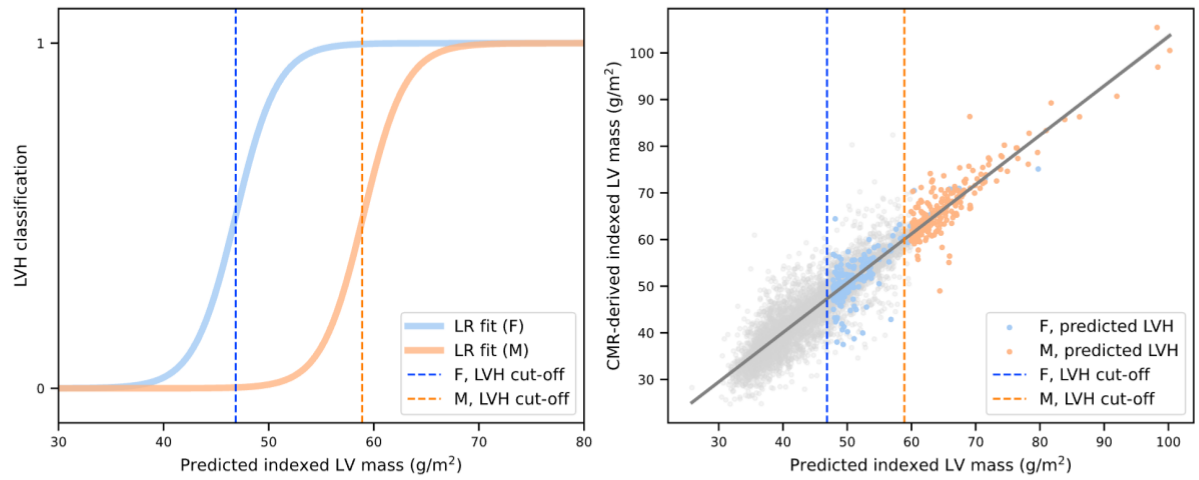

Supplementary Figure 1. LVH decision thresholds for FCN_{LVM} + LR, in terms of the LR fit (on predicted LVM values from FCN_{LVM}) and corresponding partition of the indexed LVM regression. The LVH cutoffs (vertical dashed lines) are >58.9g/m^2 for males and >46.9 g/m^2 for females, noting that both correspond to model-predicted indexed LVM as opposed to CMR-derived LVM.

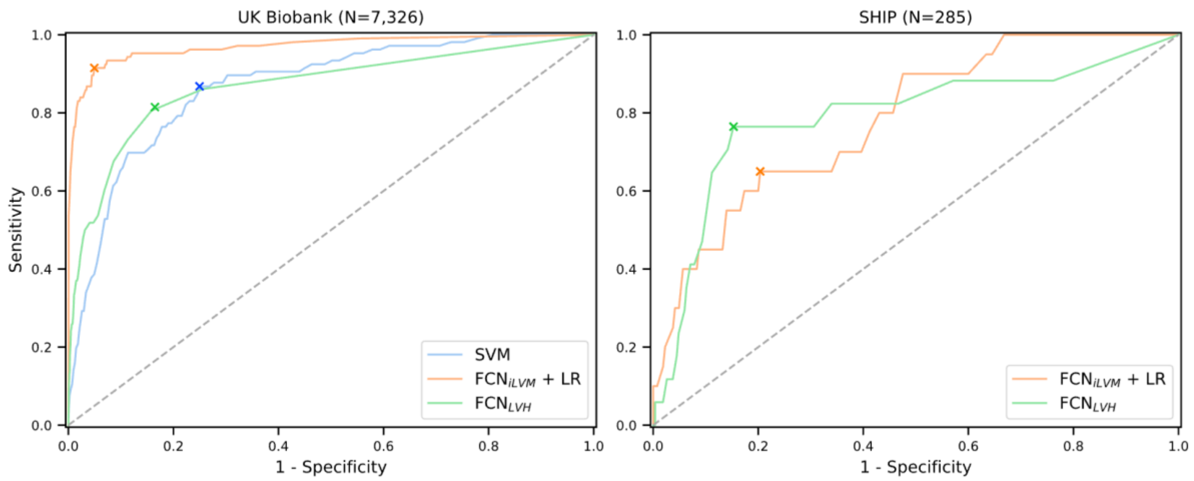

Supplementary Figure 2. Receiver operating characteristic (ROC) curves for LVH classification, in the UKB and SHIP cohorts (test sets). Curves include the benchmark SVM (replication of Naderi et al., 2023) for UKB alone, alongside the FCN_{LVH} and FCN_{LVM} + LR from the present work. The operating points for the reported sensitivity/specificity are annotated with crosses, both of which optimize Youden's J statistic.

THE EHRA AI checklist for reporting, reading and understanding AI studies in clinical EP				
**Item #**	**Category/Section**	**Explanation**	**Rationale**	**Page #**
	**TITLE**			
i)	***Title***	Include clear terms to identify the study as using artificial intelligence, machine learning or other specific terms	To facilitate paper retrieval the terms artificial intelligence/machine learning/neural network in the context of EP should be used	1
	**INTRODUCTION**			
1	**Intended clinical use**	Clearly describe the intended use and where in clinical workflow the model can be used and the objective of the study	To provide clear information of the clinical context in which to use the suggested AI solution in the context of EP	4
2	**Clinical benefit**	Added benefit of AI compared to standard clinical care (gold standard)	To explain how the AI is performing compared to clinical care (gold standard/standard practice) to better evaluate the performance of the AI model and its potential added benefit	4
	**METHODS**			
3	**Data Collection**	Describe how data was collected	To provide a clear description of the dataset generation process, for example was data retrospectively of prospectively collected, from a single center, or multicenter?	5
4	**Source (of data)**	Describe the study design or source of input data and how it was acquired	To describe how the input data was acquired including the study design - for example RCT, cohort, registry data	5
5	**Development data set (model training data set)**	Describe the data set	To describe the data set that was used for training of the model (i.e 12-lead ECGs from a specific population)	5-6
6	**Participants**	Describe the participants in the data sets, including eligibility criteria (inclusion and exclusion criteria).	Flow chart of participants (or table) suggested	5
7	**Comparator**	Provide clear definition of how the gold standard was collected.<br>Clearly describe the gold standard and ground truth including limitations.	To describe in detail how ground truth the model was trained on was established - human interaction, consensus, review type). For example, how was the diagnosis of atrial fibrillation established (12 lead ECG interpreted by independent electrophysiologists)	5
8	**Validation data set**	Describe the validation data set, in particular defining the data set split.	To describe in detail the data set that was used for validating the model, and the rationale bases on which the whole dataset was split and how.	10
9	**Sample Size**	Explain how the study size was arrived at.	For supervised models: Focus in particular on the training set including number of positives/negatives and the use of data augmentation/reduction (legitimization). For unsupervised models: focus on the number of participants	5, 10
10	**Outcome**	Clearly define standardized and reproducible outcome of clinical relevance.	To clearly describe the outcome, for example the accuracy of a specific algorithm	9

11	**Data type (source)**	Clearly describe the data type for the study, including pre-processing	To describe the data used (i.e., ECG, image, EGM, omics, EHR..) and its specification used to train and validate the model (i.e., was the information from an ECG in a image or a digital format)	5-6
12	**Data Preparation**	*Input data handling, data augmentation and selection prior to analysis by the AI system, application of techniques to prevent data leakage.*	To describe every step of handling the data (i.e., was the data reused at any time in the model, like using one ECG to provide several data points)	5-6, 8
13	**Balanced groups**	Clearly state how/if groups were balanced	To describe in detail the data set that was used for validating the model, and the rationale bases on which the whole dataset was split and how.	9
14	**Data issues (missingness / poor data / duplication / outliers)**	Describe how handling of data of poor quality/noise/missing data was performed	To provide information about possible issues in the utilized data, as well as how these were identified and handled. It should also be specified if there was a minimum standard for quality required for the input data, and where this standard was not achieved, how this was handled	10
15	**Feature engineering (extraction/ selection/reduction)**	If features are used, feature selection should be described including by whom features were extracted.	To describe the process of feature selection (i.e., handcrafted or automatically generated), as well as the strategy adopted to reduce their number (i.e., threshold on cumulative explained variance)	9 + Ref 6
	**REGULATORY**			
16	**Legal framework**	Clearly state if the software has been approved by legal authorities, e.g. Certificate of conformity (EU) or FDA approval or other, and add further details, where appropriate (e.g. risk class).	To provide information about the certification process undergone by the AI software specific version, and associated risk class for its use as declared by the manufacturer	19
17	**Explainability**	Is the AI model explainable on the patient level or on a population level.	To provide a description of the methodology used to provide model explainability	11
18	**Ethical approval**	Provide information on ethical approval of the study.	To clearly describe which entity evaluated and released the ethical approval for the study	19
19	**Fairness**	Describe inclusion of relevant groups in the dataset	To describe the efforts made to ensure fairness in the study, including for example age, ethnicity and gender	17
	**OPEN SCIENCE**			
20	**Data availability/ Code sharing**	Is the data available on a public website? Is the code available?	To provide details on how to access the anonymized data used for training/validating the model, as well as code sharing	19
21	**Trial registration**	In case of a trial, clearly state if and where the trial is registered.	Provide the number and the reference for the trial registration.	N/A
	**RESULTS**			
22	**Participants**	Baseline demographics (internal and external validation data).	*To clearly describe the participant demographics in the study/trial/inclusion to perform internal validation of the AI model, as well as the dataset used for external validation."*	11-12 + Table 1

23	**Training performance**	Provide results from the training data set	To provide results using proper metrics describing the model performance when applied to the training set, in order to provide a reference for the expected model performance and allow overfitting assessment in non-externally validated studies	Not provic
24	**Internal validation**	The results from the testing data set	To provide results using proper metrics describing the model performance when applied to the validation set, as obtained from the same population/hospital/study/equipment	12 + Table 2
25	**External validation**	The results from the external validation data set	To provide results using proper metrics describing the model performance when applied to a validation set obtained from a different population/hospital/study/equipment	13 + Table 3
26	**Model performance Internal and external validation**	Choose appropriate metric selection for reporting	"To provide appropriate metrics (threshold dependent or independent), for example: AUC/Sensitivity/Specificity/NPV/PPV/F1/Uncertainty Failing cases"	12-13 + Tables 2/3 Figure 6
27	**Performance errors**	Analysis of performance errors and how they were identified	To provide description about how errors in the model were detected, possible explanations, and potential corrections taken	12-13 + Figures 1/
28	**Performance compared to classic statistical methods**	What did the model add?	To provide a comparison with a regular statistical model if applicable, potentially using net reclassification indices (i.e., what would have been the results of a regression model compared to the AI-algorithm)"	12-13 + Table 2 + Figure 6
29	**Generalizability**	Discuss the level of generalizability of the obtained results.	To discuss how and within which limits the obtained results could be generalized to a more general population, with regards to internal and external validation data sets	15
	**CONCLUSION**			
ii)	**Conclusion**	Is the conclusion supported by the dataset?		17-18
